# CT-based radiomics models may predict the early efficacy of microwave ablation in malignant lung tumors

**DOI:** 10.1186/s40644-023-00571-w

**Published:** 2023-06-12

**Authors:** Fandong Zhu, Chen Yang, Yang Xia, Jianping Wang, Jiajun Zou, Li Zhao, Zhenhua Zhao

**Affiliations:** 1 Shaoxing University School of Medicine, Shaoxing, 312000 China; 2 Zhejiang University School of Medicine, Hangzhou, 310000 China; 3 Department of Radiology, Shaoxing Maternal and Child Health Hospital, Shaoxing, 312000 China; 4 Department of Radiology, Shaoxing People’s Hospital, Key Laboratory of Functional Molecular Imaging of Tumor and Interventional Diagnosis and Treatment of Shaoxing City, No. 568, North Zhongxing Road, Yuecheng District, Shaoxing, 312000 China

**Keywords:** Computed tomography, Early efficacy, Malignant lung tumor, Microwave ablation, Radiomics

## Abstract

**Purpose:**

To establish and validate radiomics models for predicting the early efficacy (less than 3 months) of microwave ablation (MWA) in malignant lung tumors.

**Methods:**

The study enrolled 130 malignant lung tumor patients (72 in the training cohort, 32 in the testing cohort, and 26 in the validation cohort) treated with MWA. Post-operation CT images were analyzed. To evaluate the therapeutic effect of ablation, three models were constructed by least absolute shrinkage and selection operator and logistic regression: the tumoral radiomics (T-RO), peritumoral radiomics (P-RO), and tumoral-peritumoral radiomics (TP-RO) models. Univariate and multivariate analyses were performed to identify clinical variables and radiomics features associated with early efficacy, which were incorporated into the combined radiomics (C-RO) model. The performance of the C-RO model was evaluated by the area under the receiver operating characteristic (ROC) curve (AUC), calibration curve, and decision curve analysis (DCA). The C-RO model was used to derive the best cutoff value of ROC and to distinguish the high-risk group (Nomo-score of C-RO model below than cutoff value) from the low-risk group (Nomo-score of C-RO model higher than cutoff value) for survival analysis of patients.

**Results:**

Four radiomics features were selected from the region of interest of tumoral and peritumoral CT images, which showed good performance for evaluating prognosis and early efficacy in three cohorts. The C-RO model had the highest AUC value in all models, and the C-RO model was better than the P-RO model (AUC in training, 0.896 vs. 0.740; *p* = 0.036). The DCA confirmed the clinical benefit of the C-RO model. Survival analysis revealed that in the C-RO model, the low-risk group defined by best cutoff value had significantly better progression-free survival than the high-risk group (*p*<0.05).

**Conclusions:**

CT-based radiomics models in malignant lung tumor patients after MWA could be useful for individualized risk classification and treatment.

## Key points

Radiomics features combined with clinical factors performed well in prognosis evaluation.

CT-based radiomics models can predict the survival after MWA in malignant lung tumor patients.

## Introduction

Lung cancer seriously threatens the life and health of humans, and it takes the lives of more people than any other cancer [1, 2]. Surgical intervention is most applicable to early-stage lung cancer patients and is considered the best curative option [3]. However, approximately 80% of lung cancer patients do not benefit from surgical resection because the disease is detected at a late stage and progresses rapidly [4]. In most patients with unresectable lung cancer, the benefits from traditional chemotherapy and radiotherapy are limited [5, 6]. Several novel local treatment strategies have emerged in recent years including local ablation therapy. Among various ablation therapies, microwave ablation (MWA) is one of the most effective methods for the treatment of lung neoplasms [7, 8]. How to define and judge immediate efficacy is one of the problems that must be urgently solved. The ablated lesion is usually slightly larger than at baseline because of tissue edema in the first 3 months; however, after 3–6 months, the lesion becomes smaller, and any increase in volume at this stage is considered a possible relapse [9]. Ye et al. [4] proposed that the effect of thermal ablation can only be detected after 3 months of imaging follow-up. However, there are no explicit criteria to assess post-ablation performance and early prognosis after MWA [10]. Therefore, it is necessary to identify new biological markers to evaluate efficacy before 3 months, and stratification of prognostic risks could allow more timely interventions, such as second MWA and adjustment of other therapies, which might help to reduce tumor load and prolong survival [11].

Radiomics is defined as the high-throughput mining and quantification of radiologic images, which provides essential information about the tumor and peritumor microenvironment [12, 13]. Radiomics features obtained from CT images have shown excellent diagnostic and prognostic performance in many tumors including brain cancer [14], nasopharynx cancer [15], liver cancer [16], and lung cancer [17]. Markich et al. [18] identified pre-radiofrequency ablation (RFA) features predicting local control of lung metastases following RFA. The highest prognostic performance was reached with a multivariate model including an Radiomics prognostic score built on four radiomics features from pre-RFA and early revaluation CT scans (cross-validation concordance index = 0.74) in which the Radiomics prognostic score remained an independent predictor [cross-validated hazard ratio = 3.49, 95% confidence interval (CI), 1.76–6.96]. Liu et al. [19] explored three CT-based radiomics features as prognostic factors for local tumor progression after ablation of lung cancer, and used the best threshold value for each feature to stratify prognostic risks for patients, that is, into high and low risk. To the best of our knowledge, there are no studies aimed at developing tumoral and peritumoral models to predict the early efficacy and prognosis of MWA in malignant lung tumors, also no studies that used the cutoff value of combined model that integrated tumoral, peritumoral, and clinical features to classify patients into high-risk and low-risk groups for survival analysis. Here, we used a new radiomics approach to identify tumoral and peritumoral imaging biomarkers for predicting early efficacy of MWA in malignant lung tumors [20, 21].

This study explored the non-invasive prediction of early postoperative efficacy (less than 3 months) using three models of pulmonary malignant ablation: the tumoral radiomics (T-RO), peritumoral radiomics (P-RO), and tumoral-peritumoral radiomics (TP-RO) models, and combined radiomics (C-RO) models. The predictive performance of the models were evaluated using independent testing and validation groups. The cutoff value of receiver operating characteristic (ROC) was calculated to stratify the risks of the patient prognosis.

## Materials and methods

### Patients

**Fig. 1:**
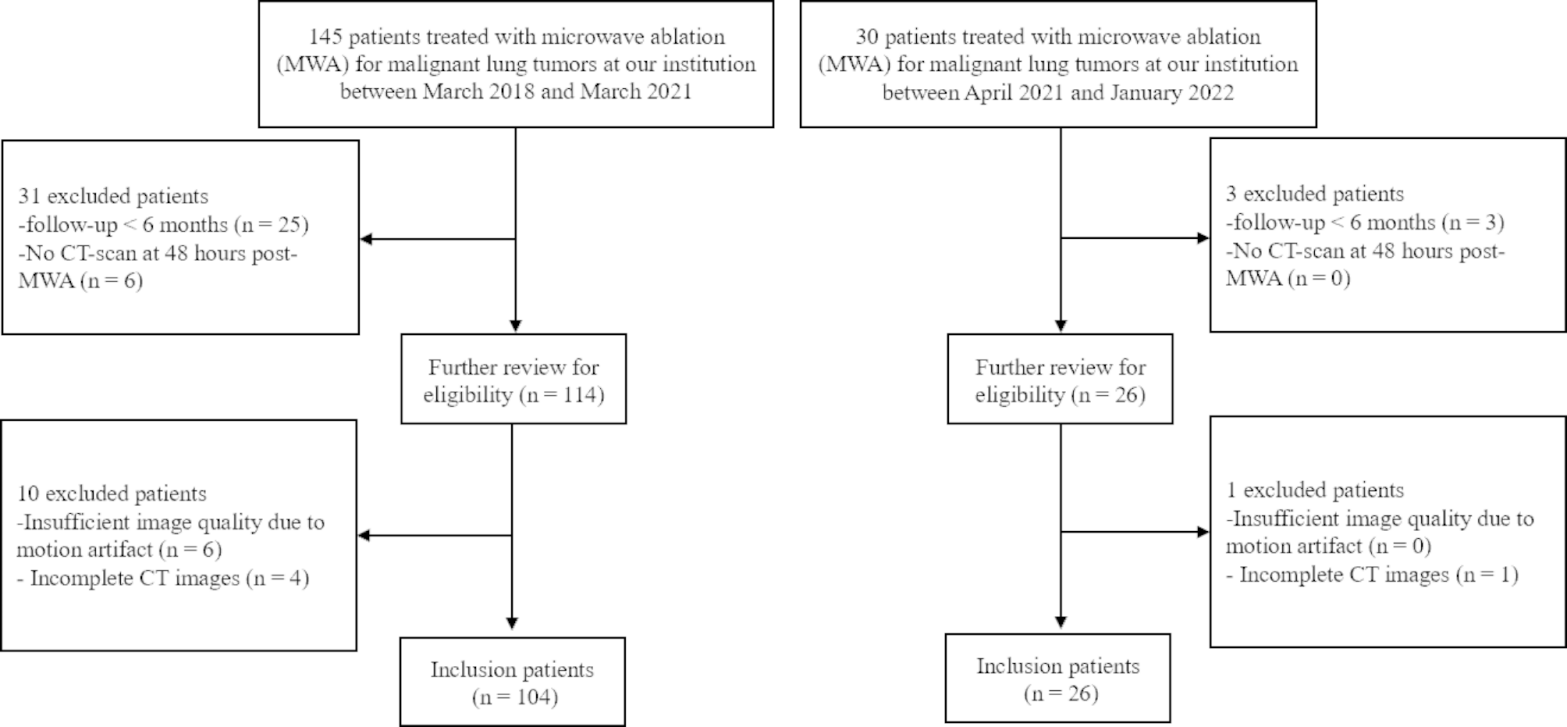
Flow diagram of the study enrolment patients

The hospital ethics committee approved this retrospective study and waived the need to sign informed consent forms. A total of 145 patients (between March 2018 and March 2021) and 30 patients (between April 2021 and January 2022) with confirmed malignant lung tumors treated with MWA in Shaoxing People’s hospital (Shaoxing, China) were enrolled in the study. All primary lung cancers and some metastatic lung cancers were confirmed by needle biopsy, and remaining tumors were presumed to be metastatic lung cancer based on imaging characteristics. CT images were collected immediately after MWA treatment in 16-slice spiral CT scanner (MinFound, Shaoxing Zhejiang, China). All patients were staged according to the modified Response Evaluation Criteria in Solid Tumors (m-RECIST) criteria [22]. Finally, a total of 104 patients of the first period were enrolled and assigned to the training and testing cohorts randomly at a ratio of 7:3 [23]. The training cohort included 72 patients and the testing cohort included 32 patients. A total of 26 patients of the next period were enrolled to the validation cohort (Fig. 1).

### Pre-ablation assessment and ablation procedure

Immediate pre-ablation imaging on the day of ablation was performed using a 16-slice spiral CT scanner (Min-Found) with the following settings: 16 × 1.2 collimation, 120 kV, 160 mAs, 0.5 s/round (s/r) and 2 mm section thickness. Two interventional radiologists, each with more than 5 years of experience, examined each lesion on CT images to plan the ablation procedure. Patients who were taking anticoagulants were required to stop taking them for 7 days prior to the ablation.

Patients were positioned in a prone or supine position for each surgery depending on the ablation method. Analgesic medication with lidocaine (1.67 mg/kg body weight) was administered. All ablations were performed using CT fluoroscopic guidance with the following parameters: 16 × 0.6 collimation, 120 kV, 80 mAs, 0.5 s/rand 2 mm section thickness.

The determined antenna entrance was established under local anesthetic immediately before start of the ablation procedure. Microwave antennas were inserted through a single pleural puncture after performing a small skin incision. Only one microwave antenna was used for ablation in each case. The energy output was adjusted to achieve complete ablation according to the manufacturer’s protocols, which vary depending on the size of the lesion. The MWA course was completed by needle-path cauterization under thermal coagulation to avoid tumor cell implantation when the tumor was completely covered by ground-glass opacity (GGO; indicating the ablation area), as verified by intraoperative CT. For few lesions adjacent to large blood vessels and bronchi, although the morphology of GGO may vary after MWA, the primary principle was to ensure complete coverage of the lesion by GGO. Patients were required to remain on bed rest for the first 8 h after ablative surgery, during which they were closely monitored by physicians.

### Endpoints and follow-up

The lesions after 3 months of MWA treatment were used as the reference [9]. According to m-RECIST criteria, the clinical treatment response after MWA was defined: Complete response (CR) refers to the disappearance of arterial enhancement in all target lesions. Partial response (PR) refers to a reduction of ≥ 30% in the total length diameter of all target lesions. Progressive disease (PD) refers to an increase of 20% in the total of the length diameter of all target lesions, or the emergence of new lesions; Stable disease (SD) means that the target lesion has neither shrunk to PR nor enlarged to PD. Patients were categorized into effective treatment (CR + PR + SD) and ineffective treatment (PD). All patients were followed-up immediately after MWA treatment, then every 3 months during the year after MWA treatment, and annually after that.

### Workflow of radiomics analysis

**Fig. 2:**
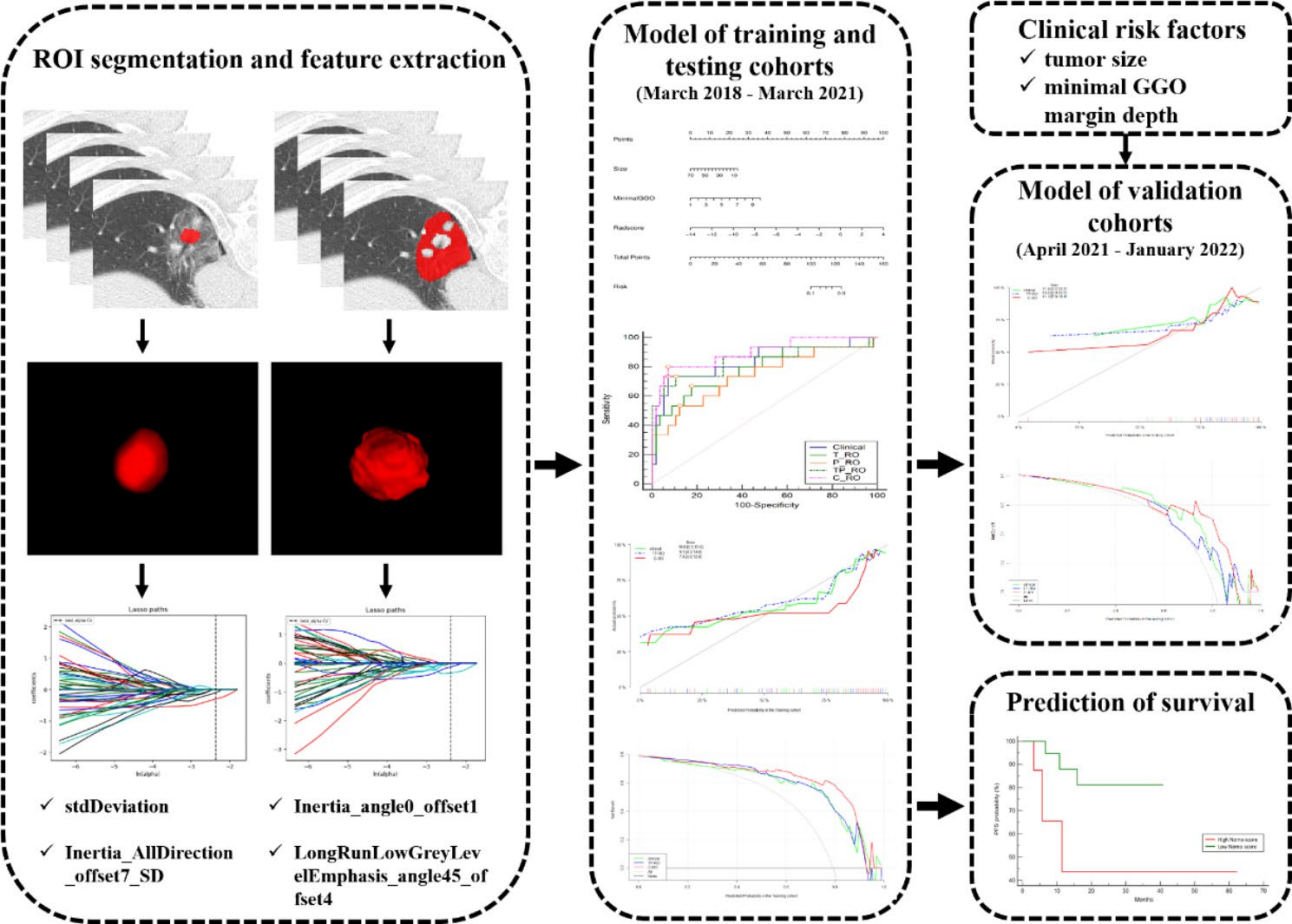
The workflow of the radiomics model construction

The workflow of the radiomics analysis included image segmentation, feature extraction, feature selection, model building and model evaluation (Fig. 2).

### Image segmentation

**Fig. 3:**
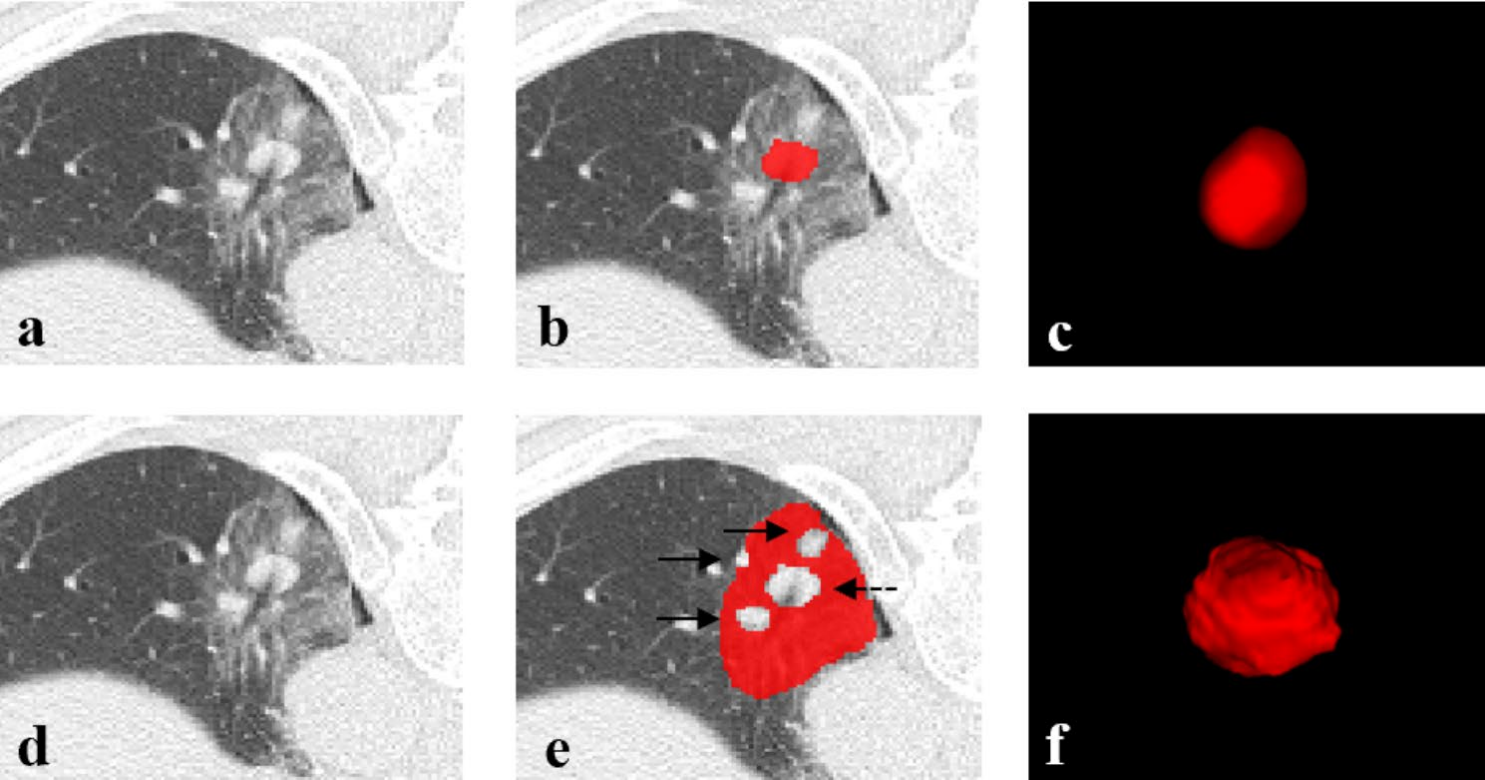
The region of interest (ROI) is depicted as a drawing. A 64-year-old man with hepatocellular carcinoma metastatic to the left lower lobe, as determined by histopathology. (**a. d**) CT image in the lung window. (**b**) The manually delineated ROI in the tumor for the T-RO model. (**c**) A 3D lesion was generated for the T-RO model. (**e**) The manually delineated ROI around the tumor for the TP-RO model. The solid black arrows indicate the large blood vessels; the black dotted arrows indicate lesions. (**f**) A 3D lesion generated for the TP-RO model

A radiologist with 10 years of experience used ITK-SNAP software (version 3.8.0, http://www.itksnap.org) to perform three-dimensional segmentation of malignant lung tumors, the radiologists were blinded to the medical history and the follow-up results. The region of interest (ROI) was manually drawn in the tumoral and peritumoral regions on CT images, avoiding large blood vessels and the bronchus. The peritumoral region is indicated by GGO around the tumor. A radiologist with 20 years of experience confirmed the segmentation results (Fig. 3).

### Radiomics feature extraction

Imaging features were calculated for each patient using Artificial Intelligence Kit software (A.K. software; GE Medical Healthcare, Milwaukee, Wisconsin). Features from tumor, peritumor, or a combination were used for subsequent analyses. A total of 396 radiomics features were extracted [24], including 42 histogram features, 180 Gy-level run-length matrix features, 19 morphological features, 11 Gy-level size-zone matrix features, and 144 Gy-level co-occurrence matrix features.

### Radiomic feature selection and model building

**Fig. 4:**
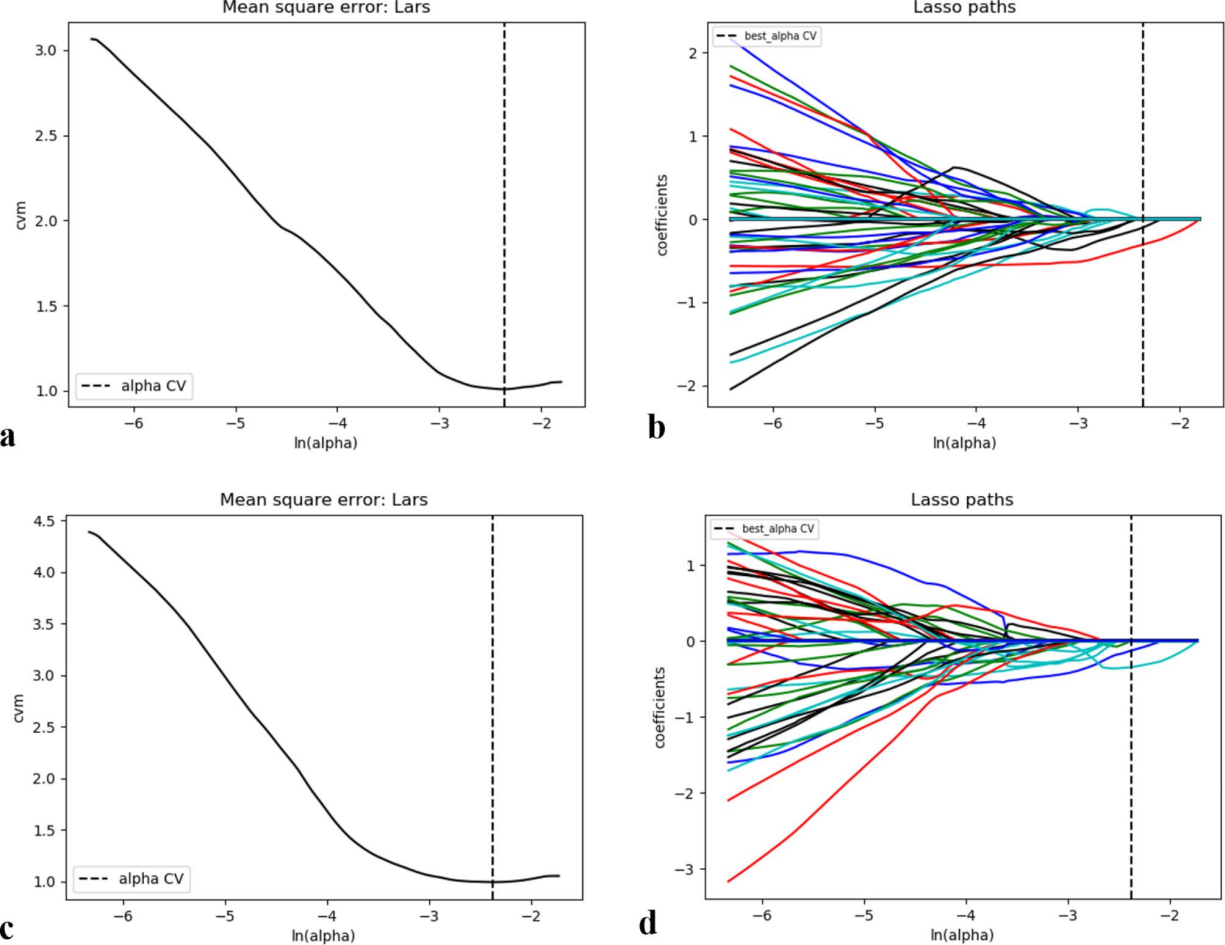
Lasso path plot of the T-RO model (**a**) and P-RO model (**c**) in the training samples. Mean square error on each fold for the lasso of the T-RO (**b**) and P-RO (**d**) models

To eliminate dimensional disparities, each image was normalized to achieve a nonzero mean and unit variance across the training, testing and validation cohorts. Both feature selection and model building were performed in the training cohort (Fig. 4).

Tumoral and peritumoral features were selected using least absolute shrinkage and selection operator (LASSO) regression via a continuous shrinking operation, thereby minimizing regression coefficients to reduce the likelihood of overfitting [25]. Single factors for determining treatment efficacy were assessed using univariate analysis. Important variables from univariate analysis were then entered into a multivariate logistic regression model to identify potential risk factors associated with treatment outcome. The fusion radiomics feature combined the tumoral and peritumoral signatures. The clinical risk factors were combined with the tumor and peritumor fusion radiomics features to build a combined model using multivariable logistic regression modeling.

### Model evaluation

AUCs were used to measure the discriminative power for predicting treatment efficacy using ROCcurves. The Delong test was used to compare the curves many times with Bonferroni-adjusted *p* values. The AUC was estimated with 95% CI, as well as sensitivity and specificity. In the combined model, a radiomics nomogram was created as a graphical representation. Harrell’s C-index was used to assess the nomogram’s discriminative ability. To analyze the agreement between nomogram-predicted treatment efficacy and actual treatment efficacy using the calibration curves, the Hosmer-Lemeshow test was performed. The net benefits at different threshold probabilities in the cohorts were measured, and DCA was used to establish the nomogram’s clinical relevance.

### Survival analysis

The disease progression was used as an endpoint in the patient survival analysis, which was defined as an increase of ≥ 20% in the total of the length diameter of all target lesions, or the emergence of new lesions. The survival curve was created by Kaplan-Meier survival analysis to assess patient survival based on radiomics features of tumoral and peritumoral ROIs. The threshold for separating patients into two survival groups was computed individually for best cutoff value in the C-RO model for all patients. For each threshold, subjects were grouped into survival cohorts above or below the selected threshold value. The *p-*value between the two survival curves was calculated.

### Statistical analysis

SPSS (version 23.0, US) was used for univariate analysis of clinical features. The “glmnet” package of R software (version 4.0.3, US) was used to perform LASSO logistic regression to select predictive radiomics features. 5-folder cross validation on training cohort was used to choose the optimal L1 regularization strength. The “rms” package was used to construct nomograms and for decision curve analysis (DCA). ROC curves were used to identify the optimal cutoff values for the Nomo-score (maximizing the sum of sensitivity and specificity) to predict high-risk and low-risk patients, and Kaplan-Meier survival analysis was used to analyze the progression-free survival (PFS) of the different risk groups. A two-tailed *p*-value < 0.05 was considered statistically significant.

## Results

### Clinical characteristics

A total of 130 patients were finally enrolled in the study, including 104 patients who were judged as effective treatment, and 26 patients who were judged as ineffective treatment cohort. The clinical characteristics are shown in Table 1. Univariate analysis showed that the factors of age, tumor size, minimum GGO margin depth (The minimum distance between the tumor margin and the ablation zone margin), adjacent large bronchus were significantly related to effective treatment and ineffective of MWA (Table 1).

**Table 1:** Comparisons of patient characteristics

Characteristics	Effective treatment cohort	Ineffective treatment cohort	*p* value
Age, year	63.27 ± 10.73	69.38 ± 10.05	0.010^*^
Gender			0.096
Male	49 (47.1%)	17 (65.4%)	
Female	55 (52.9%)	9 (34.6%)	
Tumor pathology			0.387
Primary	20 (19.2%)	7 (26.9%)	
Metastasis	84 (80.8%)	19 (73.1%)	
Tumor indicators			0.927
Absent	37 (35.6%)	17 (65.4%)	
Present	67 (64.4%)	9 (34.6%)	
Systematic treatment			0.093
Absent	66 (63.4%)	21 (80.8%)	
Present	38 (36.5%)	5 (19.2%)	
Tumor Size	12.50 (9.03, 17.15)	20.05 (12.53, 31.83)	<0.001^*^
Minimum GGO margin depth	5.93 ± 1.98	4.78 ± 1.82	0.008^*^
Maximum GGO margin depth	11.95 (9.75, 15.88)	11.30 (8.38, 15.73)	0.331
Density			0.558
Solid	89 (85.6%)	24 (92.3%)	
Non-Solid	15 (14.4%)	2 (7.7%)	
Location			0.725
Upper middle lobe	56 (53.8%)	13 (50.0%)	
Lower lobe	48 (46.2%)	13 (50.0%)	
Adjacent large blood vessels			0.088
Absent	67 (64.4%)	12 (46.2%)	
Present	37 (35.6%)	14 (53.8%)	
Adjacent large bronchus			0.001^*^
Absent	96 (92.3%)	17 (65.4%)	
Present	8 (7.7%)	9 (34.6%)	
Margin			0.065
Smooth	72 (69.2%)	13 (50.0%)	
Rough	32 (30.8%)	13 (50.0%)	
Pleural pull			0.170
Absent	26 (25.0%)	16 (61.5%)	
Present	78 (75.0%)	10 (38.5%)	

*p*-value reflected the differences between the effective treatment and ineffective treatment cohort, ^*^p < 0.05 (two-sided) was considered statistically significant. Minimum (Maximum) GGO margin depth refers to the minimum (maximum) distance between the tumor margin and the ablation zone margin

In the multivariate analysis, the factors of tumor size [odds ratio (OR) 0.919, 95% CI 0.865–0.976] and minimum GGO margin depth (OR 1.353, 95% CI 1.013,1.807) were independent predictors of MWA between effective treatment and ineffective treatment (Table 2).

**Table 2:** Multivariate analysis of patient characteristics

Characteristics	B	OR (95%CI)	*p* value
Age	-0.037	0.964 (0.918, 1.012)	0.137
Tumor Size	-0.085	0.919 (0.865, 0.976)	0.006^*^
Minimum GGO margin depth	0.302	1.353 (1.013, 1.807)	0.041^*^
Adjacent large bronchus	1.057	2.878 (0.854, 9.696)	0.088

^*^p < 0.05 (two-sided) was considered statistically significant. Minimum GGO margin depth refers to the minimum distance between the tumor margin and the ablation zone margin

### Radiomics characteristics

The training cohort included 72 patients (57 effective treatment vs. 15 ineffective treatment), the testing cohort included 32 patients (26 effective treatment vs. 6 ineffective treatment), and the validation cohort included 26 patients (21 effective treatment vs. 5 ineffective treatment). A total of 396 imaging features were finally calculated for each patient from the extracted tumoral or peritumoral regions of CT images. Four features were finally selected from the combination of the two modalities. The comparison of radiomic characteristics is shown in Table 3. Statistically significant differences between the effective and ineffective treatment of MWA in the training cohorts were found in stdDeviation (*p* = 0.017), Inertia_AllDirection_offset7_SD (*p* = 0.037), Inertia_angle0_offset1 (*p* = 0.011), and LongRunLowGreyLevelEmphasis_angle45_offset4 (*p* = 0.039,Table 3).

**Table 3:** Comparisons of radiomics features between the training, testing and validation cohorts

Different models		Training cohort (n = 72)			Testing cohort (n = 32)			Validation cohort (n = 26)		
		Effective treatment cohort	Ineffective treatment cohort	*p* value	Effective treatment cohort	Ineffective treatment cohort	*p* value	Effective treatment cohort	Ineffective treatment cohort	*p* value
T-RO	stdDeviation	-0.166 (-1.096, 0.616)	0.584 ± 1.089	0.017^*^	0.272 ± 1.127	1.357 ± 0.854	0.035^*^	0.054 ± 1.068	-0.227 ± 0.844	0.590
	Inertia_AllDirection_offset7_SD	-0.266 (-0.289, -0.243)	-0.251 (-0.266,0.949)	0.037^*^	-0.212 (-0.298, 0.315)	-0.009 (-0.266, 0.971)	0.356	-0.314 (-0.314, -0.233)	-0.114 (-0.332, 2.415)	0.477
P-RO	Inertia_angle0_offset1	-0.389 (-0.797, 0.315)	0.731 ± 1.344	0.011^*^	-0.330 ± 1.063	0.747 ± 1.173	0.036^*^	-0.816 ± 0.949	0.343 ± 1.345	0.414
	LongRunLowGreyLevelEmphasis_angle45_offset4	-0.307 (-0.513, -0.142)	-0.208(-0.396,1.065)	0.039^*^	-0.320 (-0.505, -0.121)	-0.374 (-0.461, 0.753)	0.772	-0.253 (-0.918, 0.314)	0.192 ± 0.887	0.454

^*^p < 0.05 (two-sided) was considered statistically significant

### Performance of the radiomics models

**Fig. 5:**
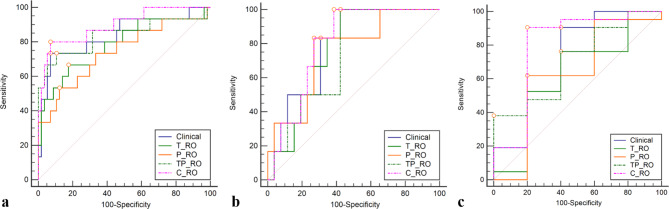
Receiver operating characteristic (ROC) curves of the four models in the training cohort (**a**) and validation cohort (**b**). The blue, green, and orange solid lines represent the Clinical, T-RO, and P-RO models, respectively. The green and purple dotted line represent the TP-RO and C-RO models. In the training cohort, the P-RO and C-RO models were considered statistically significant (*p* = 0.036)

The ROC curves of the three radiomics models are shown in Fig. 5. The AUC, sensitivity and specificity of the TP-RO model were higher than that of the T-RO model or the P-RO model in the training cohorts (Table 4).

**Table 4:** Comparisons of models in the training, testing and validation datasets

Different models	Training cohort (n = 72)			Testing cohort (n = 32)			Validation cohort (n = 26)		
	Sensitivity	Specificity	AUC (95% CI)	Sensitivity	Specificity	AUC (95% CI)	Sensitivity	Specificity	AUC (95% CI)
Clinical	91.2%	73.3%	0.839(0.718,0.942)	76.9%	50.0%	0.750(0.563,0.917)	90.5%	60.0%	0.724(0.515,0.879)
T-RO	80.7%	66.7%	0.784(0.647,0.901)	61.5%	83.3%	0.763(0.607,0.900)	76.2%	60.0%	0.600(0.391,0.785)
P-RO	86.0%	53.3%	0.740(0.589,0.873)	80.8%	33.3%	0.756(0.568,0.929)	61.9%	80.0%	0.629(0.418,0.808)
TP-RO	87.7%	73.3%	0.836(0.698,0.951)	69.2%	50.0%	0.731(0.552,0.885)	76.2%	60.0%	0.686(0.475,0.852)
C-RO	91.2%	80.0%	0.896(0.800,0.969)	73.1%	83.3%	0.801(0.650,0.923)	85.7%	80.0%	0.790(0.587,0.924)

AUC = area under curve

### Establishment of the C-RO model nomogram

**Fig. 6:**
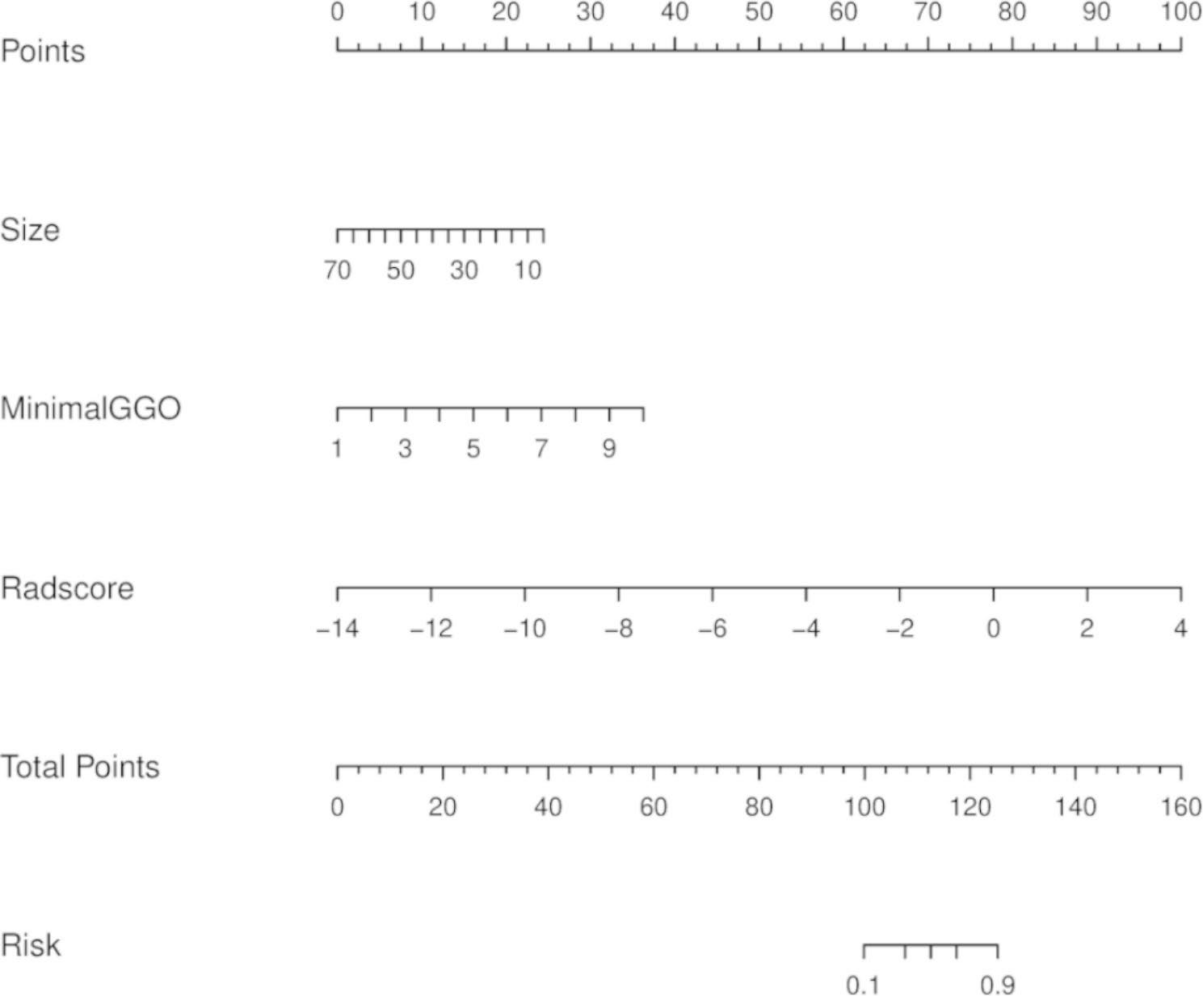
Nomogram of the C-RO model. Size = Tumor size. MinimalGGO = Minimum GGO margin depth

We developed the C-RO model nomogram (Fig. 6) that integrated the two independent clinical factors and four radiomics features.

**Fig. 7:**
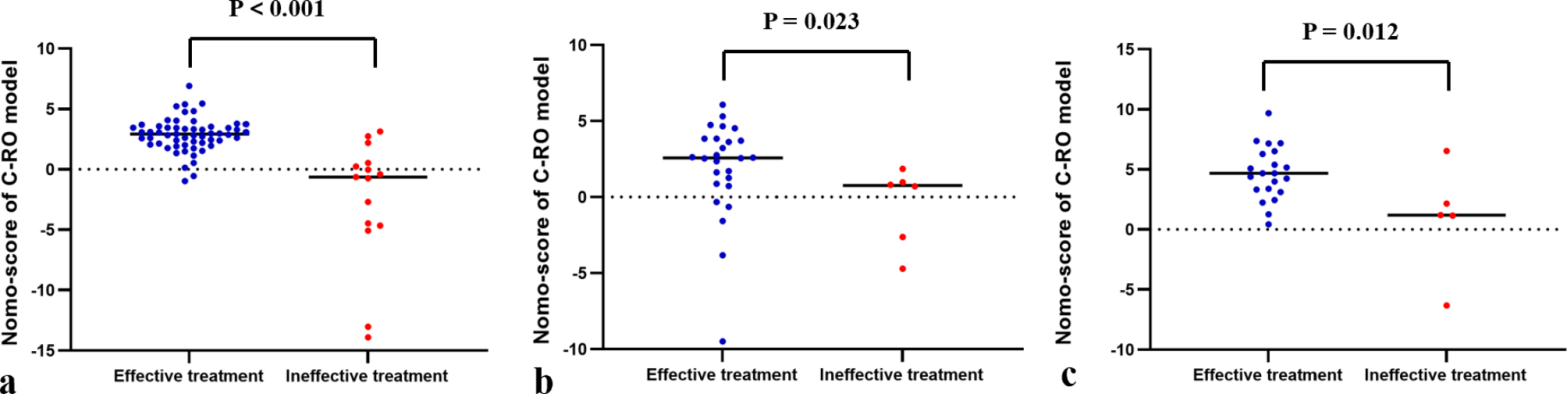
Scatter Chart of Nomo-score in C-RO model. (**a**) Training cohort. (**b**) Testing cohort. (**c**) validation cohort

The Nomo-score was calculated using the following formula: Nomo-score = 2.1625 − 0.0650 × Tumor Size + 0.6976 × Minimum GGO margin depth + 0.9617 × Rad-score. In the training, testing, and validation cohort, the Nomo-score of the C-RO model was higher in the effective treatment cohort than in the ineffective treatment cohort (*p* < 0.05, Fig. 7). The C-RO model nomogram provided the highest C-index of 0.896 (95% CI, 0.800–0.969) in the training cohort, 0.801 (95% CI, 0.650–0.923) in the testing cohort, and 0.790 (95% CI, 0.587–0.924) in the validation cohort (Fig. 5).The sensitivity and specificity of the C-RO model performed well in the training, testing and validation cohorts. Figure 5 show showed the ROC curves.

**Fig. 8:**
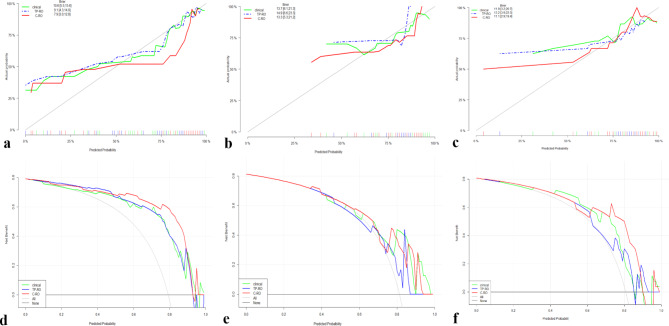
Calibration curves and decision curve analysis (DCA) of the C-RO model. Calibration curves in the training cohort (**a**), testing cohort (**b**) and validation cohort (**c**). DCA in the training cohort (**d**), testing cohort (**e**) and validation cohort (**f**). The green, blue, and red lines represent the Clinical, TP-RO, and C-RO models, respectively

In the training, testing, and validation cohorts, the calibration curves of the C-RO model nomogram demonstrated good agreement between observed actual response probabilities and nomogram projected probabilities (Fig. 8a, b and c, respectively). The Hosmer-Lemeshow test yielded nonsignificant differences (*p* = 0.856), suggesting that there was no departure from a perfect fit. The DCA for the radiomics nomogram in the training, testing and validation cohorts showed a superior net clinical benefit of the C-RO model over other models (Fig. 8d, e and f, respectively).

### The role of radiomics features in the prediction of survival

**Fig. 9:**
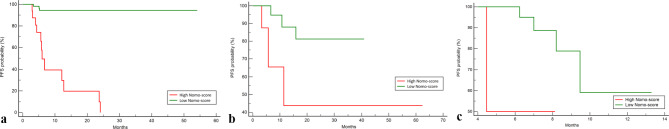
The survival curve plotted for patients with Nomo-score above or below a Nomo-score threshold of the C-RO model in the training cohort (**a**) testing cohort (**b**) and validation cohort (**c**). The red line represents the high-risk group; thegreen line represents the low-risk group

In the survival study, the median duration of follow-up was 16.3 months (range, 2.9 to 62.4), the median progression-free survival was 13.6 months (95% CI, 3.0 to 51.9). The best cutoff point based on maximizing the sum of sensitivity and specificity was a Nomo-score of 0.759 in the C-RO model. The log-rank test was used to select the Nomo-score with significance, and the best cutoff value was used to categorize patients into high-risk (Nomo-score of C-RO model below than cutoff value) and low-risk (Nomo-score of C-RO model higher than cutoff value) categories for PFS. PFS was considerably longer in low-risk group than in high-risk group (Fig. 9).

## Discussion

The aim of this study was to evaluate tumoral and peritumoral radiomics-based models and their potential to predict the early efficacy of MWA in malignant lung tumors. The model constructed by integrating radiomics features extracted from the ROI in tumoral and peritumoral areas and clinical risk factors showed a stable and excellent predictive performance, providing a non-invasive and repeatable method to evaluate the outcomes of MWA. The Nomo-score was also helpful for the analysis of local progression.

Tumors may extend 6–8 mm into the adjacent lung parenchyma, which suggests that the ablation site should be at least 1 cm larger than the lesion [26]. However, this area is seldom visible to the naked eye on ordinary CT images. Therefore, we need to explore tumoral and peritumoral ROI to predict early efficacy after MWA. In this work, we identified radiomics features on tumoral and peritumoral CT images that were capable of predicting the early efficacy of MWA for malignant lung tumors with high sensitivity. In addition, we developed, tested and validated the C-TO model, which combined clinical risk factors with radiomics features from tumoral and peritumoral CT images. The model showed good sensitivity and specificity for predicting the early efficacy of MWA. Akinci et al. [27] found that a combination of the tumoral and peritumoral radiomics signature with the TNM staging system outperformed TNM staging alone for individualized recurrence risk estimation in patients with surgically treated NSCLC. In this study, the AUC of the C-RO model was 0.896 (95% CI 0.800–0.969), which should enable clinicians to be more objective and personalized in evaluating the early efficacy of MWA in patients with malignant lung tumors and to design treatment strategies.

The radiomics of ROI extracted from tumor included stdDeviation and Inertia_AllDirection_offset7_SD. The stdDeviation is the characteristic of histogram parameters. It was reported that stdDeviation is correlated with tumor heterogeneity in lung adenocarcinoma [28]. In the training, testing and validation cohorts in this study, stdDeviation of the ineffective treatment was higher than that of the effective treatment (*p* < 0.05), which may further reflect that the tumor lesions in the ineffective treatment had higher heterogeneity after MWA. Inertia belongs to the gray level co-occurrence matrix, which reflects the sharpness of the image and the depth of the texture grooves. The low value of grooves results in low contrast and blurred images. Deng et al. [29] studied the blood supply of early lung adenocarcinomas in mice and the tumor - supplying vessel relationship. They found weak negative correlations between the solid component size of the tumor and inertia. In this study, we found that the inertia of the effective treatment was lower than that of the ineffective treatment in the training, testing and validation cohorts; this suggests that the tumoral images after MWA were fuzzy and the lesion was completely ablated, which was beneficial to the prognosis of patients. The radiomics of ROI extracted from peritumor included Inertia_angle0_offset1 and LongRunLowGreyLevelEmphasis_angle45_offset4. Inertia was higher in the ineffective treatment, which prompts higher image resolution, and the result demonstrated that ineffective treatment had incomplete ablation and residual micro-infiltrated tissue of the malignant lung tumor after MWA. LongRunLowGreyLevelEmphasis measures the simultaneous distribution of long run and low grey values. Martens et al. [30] found that LongRunLowGreyLevelEmphasis, as one of the radiomics features based on PET-CT, could predict the prognosis of patients with head and neck squamous cell carcinoma, and the larger the value, the more heterogeneous the tumor. In the early stage of malignant lung tumors after MWA, the GGO around the tumor is mainly composed of “two layers” indicating different pathological changes. The inner layer represents coagulative necrosis caused by acute thermal damage to micro-infiltrated tumor tissue; the outer layer represents hyperemia and the inflammatory reaction of normal lung tissue. The boundary between the two layers is unclear and extends into each layer. In this study, the value of LongRunLowGreyLevelEmphasis was higher for ineffective treatment than for effective treatment, this suggests that the peritumor heterogeneity of the inner layer was still present and was not completely thermally ablated.

We evaluated the clinical risk factors and performed radiological analysis. The results were consistent with previous findings that tumor size and minimum GGO margin depth are independent variables associated with the early efficacy of MWA in lung malignant tumors. Patients with large tumors have a lower survival rate after MWA than those with small tumors [31–33], and the tumor size threshold is 2–3 cm. In this study, the mean tumor sizes associated with effective and ineffective treatment were 12.5 and 20.1 mm, respectively; this was consistent with previous reports. The minimum GGO margin depth is another important prognostic factor. In this study, the minimum GGO margin depth in the effective treatment was larger than that in the ineffective treatment ( 5.93 vs. 4.78, *p*<0.01), indicating that MWA could cover tumors effectively. A study [34] by De Baere found that the ratio of post-treatment GGO to the pre-treatment tumor area is an indicator of the efficacy of treatment. A ratio > 4 indicates that the complete ablation rate at 4 months is 96% and the success rate is 61%. In this study, the ratio was larger in the effective treatment than in the ineffective treatment, although the specific values need to be further explored.

The survival curve provided an intuitive way to evaluate the early efficacy of MWA in lung tumor patients, which were divided into high-risk and low-risk groups. The survival curves of patients above the Nomo-score threshold in the C-RO model were considerably different from those below the Nomo-score threshold in the training, testing and validation cohorts. The survival curve suggests that patient survival depends on a combination of tumoral and peritumoral heterogeneity and radiomics manifestations of disease progression after MWA. Although the current study was limited by a relatively small patient cohort, it showed the potential utility of radiomics features for survival modeling with specific features and threshold values determined by the patient cohort.

The present study had several limitations. First, this was a retrospective study conducted at a single center, and the inherent bias may have affected the results. We recommend complementing our database with prospective validation using bigger cohorts from additional locations. Second, the small sample size made it difficult to conduct subgroup analysis in patients with primary and metastatic tumors; it was also difficult to analyze subgroups of patients with or without systematic treatment, additional studies should be performed to conduct subgroup analysis in the future. Although this study provided initial evidence that the T-RO, P-RO, TP-RO, and C-RO models can help predict the early efficacy of MWA in malignant lung tumors, additional prospective studies should be performed to validate the present results.

## Conclusion

Differences in radiomics features predicted the early efficacy of MWA in malignant lung tumor patients. The T-RO, P-RO, TP-RO, and C-RO models provided prognostic information for MWA patients and may serve to design treatment strategies. The C-RO model was the most effective model. Additionally, the best cutoff value of the C-RO model provided statistically significant discrimination between the low-risk group and high-risk group of the survival curve, suggesting survival trends correlated with radiomics values. Such quantitative radiomics prognostic models of malignant lung tumors may be helpful in precision medicine and affect the design of treatment strategies.

## Data Availability

The datasets used and/or analyzed during the current study are available from the corresponding author upon reasonable request.

## Abbreviations

MWA: Microwave ablation
GGO: Ground-glass opacity
LASSO: Least absolute shrinkage and selection operator
T-RO: Tumoral radiomics model
P-RO: Peritumoral radiomics model
TP-RO: Tumoral-peritumoral radiomics model
C-RO: Combined radiomics
ROC: Receiver operating characteristic
DCA: Decision curve analysis
ROI: Region of interest
AUC: Area under the ROC curve
PFS: Progression-free survival
S/R: Seconds/round
